# C-753-07. The First Reader Study of Burn Wounds with Predictive Artificial Intelligence Analysis

**DOI:** 10.1093/jbcr/irag033.027

**Published:** 2026-04-06

**Authors:** Alisa Savetamal, Jeffrey E Carter, James Hwang, J Michael DiMaio

**Affiliations:** Bridgeport Hospital/Yale New Haven Health, Bridgeport, CT; University Medical Center Burn Center, Louisiana State University Health Sciences Center, New Orleans, LA; University of Alabama Birmingham, Birmingham, AL; Baylor Scott & White Health, Plano, TX

## Abstract

**Introduction:**

Burns are complex injuries whose severity and potential for wound healing are challenging to assess. No widespread diagnostic technology effectively predicts burn wound healing. A potential solution is multispectral imaging (MSI) processed with artificial intelligence (AI) algorithms. The primary objective of this study was to determine the impact of MSI/AI on clinicians’ treatment decisions.

**Methods:**

This study enrolled 100 independent readers (54 emergency medicine physicians (EM) and 46 burn surgeons (BS)) evaluating an identical set of burn wound images. A mixed model predicting optimal treatment assignment incorporated practitioners’ treatment decisions (surgical debridement versus conservative management) for 10 different healing and non-healing burn wounds based on digital images. Participants were then provided MSI/AI output highlighting non-healing areas overlying these digital images and were asked to (re-)assign treatment. A composite score (Clinical Accuracy Score (CAS)) of each practitioner’s treatment decisions and their confidence in the decision was calculated to determine the probability of confident and correct assessment before and after MSI/AI evaluation.

**Results:**

The probability of making a correct assessment was 61% before viewing the MSI/AI output and increased to 89% afterwards. The CAS score significantly improved from 46.6 + - 34.5 to 69.1 + - 32.2 after MSI/AI review, suggesting readers were more likely to be confidently correct (*p*<.0001). We found no significant differences between among EM and BS CAS scores before or after MSI/AI review (*p*=.53).

**Conclusions:**

MSI/AI assessment of burn wound significantly improved the accuracy and confidence of physician assessment of burn wound healing potential.

**Applicability of Research to Practice:**

MSI/AI may be a valuable tool for burn wound assessment to improve diagnostic accuracy compared to traditional methods. This technology may help clinicians standardize burn wound evaluation across providers and settings, reducing unnecessary transfers and hospital stays while offering more prudent surgical intervention.

**Funding for the study:**

This project is being supported in whole or in part with federal funds from the U.S. Department of Health and Human Services (DHHS); Administration for Strategic Preparedness and Response (ASPR); Biomedical Advanced Research and Development Authority (BARDA), under contract number 75A50123C00049. The findings and conclusions have not been formally disseminated by the DHHS and should not be construed to represent any agency determination or policy.

